# The Paratuberculosis Paradigm Examined: A Review of Host Genetic Resistance and Innate Immune Fitness in *Mycobacterium avium* subsp. *Paratuberculosis* Infection

**DOI:** 10.3389/fvets.2021.721706

**Published:** 2021-08-13

**Authors:** Amanda Kravitz, Kevin Pelzer, Nammalwar Sriranganathan

**Affiliations:** ^1^Department of Biomedical Sciences and Pathobiology, Center for One Health Research, Virginia-Maryland College of Veterinary Medicine, Virginia Polytechnic Institute and State University, Blacksburg, VA, United States; ^2^Department of Large Animal Clinical Sciences, Virginia-Maryland College of Veterinary Medicine, Virginia Polytechnic Institute and State University, Blacksburg, VA, United States

**Keywords:** Johne's disease, single nucleotide polymorphisms, *Mycobacterium avium* subsp. *paratuberculosis*, innate immunity, host resistance and susceptibility, genome-wide association study, livestock

## Abstract

Paratuberculosis, or Johne's Disease (JD) is a debilitating chronic enteritis mainly affecting ruminants caused by *Mycobacterium avium* subsp. *paratuberculosis* (MAP). This organism causes worldwide economic losses to the livestock industry, and is of public health importance due to the potential zoonotic risk between MAP and Crohn's disease (CD) in humans. Without economical treatments, or a vaccine capable of preventing infection without causing cross-reactions with bovine tuberculosis, test-and-cull methods for disease control are imperative. Unfortunately, difficulties in diagnostics and long subclinical stage hinder adequate control and is further complicated by variation in MAP exposure outcome. Interestingly, the majority of infections result in asymptomatic presentation and never progress to clinical disease. One contributing factor is host genetics, where polymorphisms in innate immune genes have been found to influence resistance and susceptibility to disease. Candidate genes identified across studies overlap with those found in CD and tuberculosis including; Solute carrier family 11 member 1 gene (*SLC11A1)*, Nucleotide-binding-oligomerization domain containing gene 2 (*NOD2*), Major histocompatibility complex type II (*MHC-II*), and Toll-like receptor (*TLR*) genes. This review will highlight evidence supporting the vital role of these genes in MAP infection outcome, associated challenges, and implications for the future of JD research.

## Introduction

Johne's disease (JD), also known as Paratuberculosis, is a chronic gastroenteritis of worldwide prevalence caused by the intracellular bacterium *Mycobacterium avium* subsp. *paratuberculosis* (MAP). Disease is responsible for severe economic losses in agriculture from infected dairy cattle, sheep, and goats. Although primarily affecting domestic and wild ruminants, MAP exhibits a range of species infectivity and has been isolated from a diverse array of non-ruminant species including rodents, birds, and rabbits (1). This disease has importance to public health due to the documented but unconfirmed link between MAP and Crohn's Disease (CD) in humans (2, 3). Transmission occurs through the fecal-oral route via ingestion of food, water, or the dam's milk to offspring contaminated with fecal material harboring MAP. The additional transmission via bodily fluids, *in-utero*, and inhalation of aerosols has been recently documented (4–7). Symptoms of clinical disease differ between species, with cattle progressing to characteristic chronic diarrhea that is absent in small ruminants. However, in all species clinical disease results in untreatable chronic granulomatous enteritis of the intestines culminating in severe progressive weight loss, and decreased milk production leading to either death or premature culling of animals. Within an infected farm, continuous MAP exposure to the herd occurs from infected animals shedding MAP in feces leading to environmental contamination. As a robust organism, MAP is capable of surviving in soil and freshwater for upwards of a year, making even one infected animal a liability to the entire herds' health (8, 9). Unfortunately, none of the current diagnostic tests are capable of identifying infected animals before fecal shedding begins, leaving asymptomatic hosts to continue transmission. The chronic nature of MAP infection and variation in host responses are further complicated by changes in diagnostic sensitivities and specificities dependent stage of infection and the individual host. For example, serum antibody enzyme linked immunosorbent assay (ELISA) has low sensitivity at early stages, but can have high sensitivity at late stages of disease. Fecal culture and fecal quantitative polymerase chain reaction (qPCR) can have low to moderate sensitivity at early stages of disease, but varies greatly by individual due to the transient nature of fecal shedding (10). These complications can cause misclassification of infected animals, especially when tests are done at only one timepoint. Differences in diagnostic tests used in genetic association studies have a direct influence on the results and is one factor influencing the lack of agreement between genetic studies of JD.

Both the microbiological characteristics of MAP, as well as the complex immunological response to infection has hindered a clear understanding of this disease, and has led to the silent spread of JD across the world. Similar to other mycobacterial pathogens, MAP replicates slowly within host monocytes and antigen presenting cells (APCs) allowing for bacterial persistence resulting in chronic infection. These asymptomatically infected animals sporadically shed MAP in feces during the 1–7 year incubation period, where they remain undetectable due to limitations of current diagnostics (10). Further complicating JD research, are the variations in host responses to MAP infection in both experimental and natural infections. This variation is documented between individuals within a population, as well as temporally and spatially within the same host (7). In fact, approximately only 10% of MAP infections progress to clinical disease while 90% remain asymptomatic (7, 10, 11). Variations in infection outcome to the causative agent of human tuberculosis, *Mycobacterium tuberculosis*, is well-documented to globally infect every 3rd human being. Worldwide estimates report that 2 billion people are infected with *M. tuberculosis*, yet only 20–30 million cases of active disease are reported annually (12, 13). Susceptibility and resistance to tuberculosis and another mycobacterial disease leprosy, has been documented and associated candidate genes for susceptibility have been implicated (14–18). Interestingly, some candidate genes proposed for tuberculosis and leprosy overlap with those identified for predisposition to Crohn's Disease (CD) in humans (19). Although factors including host age, number of exposures, dose per exposure, nutritional status, and geographical location can influence infection outcome, these alone are unable to explain the extreme variations in outcomes for both diseases. This suggests a more complex disease dynamic, where various host genetic polymorphisms affect the immunopathology between individuals that progress to either disease or remain asymptomatic within a population (13, 20–22).

In the case of JD, investigations into genetic resistance/susceptibility originated from observations of naturally infected herds with different breeds of cattle, where some breeds seemed to be resistant to MAP infection compared to others (23). Breed differences in the occurrence of JD has also been documented in sheep, goats, and red deer (24–26). Although not definitive evidence for varied genetic resistance, these breed observations led to heritability estimates using known genetically related animals to better understand the genetics behind this disease. Heritability estimates for a population are calculated based on the proportion of phenotypic variation due to genetic variation, and result in values between 0 and 1 (27). On average, heritability estimates for susceptibility to JD in dairy cattle worldwide are reported to be low to moderate with values between 0.01 and 0.283 (28, 29). This low to moderate range of heritability estimates indicate resistance/susceptibility to JD is partially influenced by host genetics, and provide evidence for a complex polygenic influence on disease outcome. Thus, selective breeding for resistance to disease may be a method to enhance current control and prevention to reduce incidence of this devastating disease.

Recent advances in animal genetics have enabled candidate gene, and Genome Wide Association Studies (GWAS) to shed light on the impact of host genetics on MAP infection. Genomic sequencing technologies such as High-Throughput sequencing (HTS) and Next-Generation sequencing (NGS) have built upon the classical chain termination Sanger method, and have allowed for higher sequencing depths and the ability to discover novel variants (30). This new technology has allowed for easier identification of genomic markers including SNPs and generation of high-density SNP microarrays, providing genome-wide data for thousands of SNPs on multiple individuals on a single microarray (31). Genetic association studies using these technologies aim to identify variations in genotype representative of a phenotype, in this case resistance/susceptibility to disease, leading to a better understanding of disease mechanisms.

Differences in host genotypes influence resistance to disease through potential effects on gene regulation, expression, and complex interactions with other genes that can lead to differences in protein function. Although complicated by environmental factors, investigations on genetic variations such as SNPs and microsatellite polymorphisms have the ability to propel the field of paratuberculosis forward by illuminating host differences that may correspond to JD resistance. Gaining insight into the roles and markers associated with resistance/susceptibility will aid in selective breeding for resistance to this disease, which together with current control strategies could lead to a significant decrease in the presence of MAP infection in domestic ruminants. This work expands upon the studies reviewed a decade ago by Purdie et al. (32), and includes recent publications with a focus on evidence for the vital role of innate immune fitness in early MAP infection. This article will review the current literature on host genetic influence on MAP infection and JD outcomes with a focus on the continuously proposed candidate genes *SLC11A1, NOD2, TLRs, MHC-II*, and their function in the innate immune response to MAP infection. Literature chosen for this review found evidence of association to MAP infection in cattle, sheep, and goats where more than one species had evidence of a specific target gene, that also overlapped with evidence from CD association studies. GWAS and GSEA-SNP studies included highlighted the interconnected pathways associated with MAP infection across species to highlight areas for future translational research. Limitations associated with genetic association studies, disease specific challenges, and their influence on study results will also be discussed. The above will be evaluated and discussed in terms of benefiting future directions for advancement in paratuberculosis research, and the impacts these advancements may have on veterinary medicine and public health.

## Candidate Gene Studies

Candidate gene studies are used to investigate if previously identified variants in genes associated with resistance/susceptibility holds true in related diseases. For example, intracellular bacterial infections like salmonellosis, brucellosis, tuberculosis, and leprosy have been found to share host genetic variants associated with susceptibility to infection (33–35). The first indications of host genetic influence on susceptibility to intracellular bacterial diseases derive from experimental studies in mice and have progressed to studies in natural hosts including humans and ruminants. The majority of candidate genes investigated in terms of JD are required for innate immune activation, signaling, and subsequent development of adaptive immunity. Results from candidate gene studies vary depending upon multiple factors, including the population studied and diagnostic tests used. Despite differences between studies, there is agreement in proposed candidate genes seen across breeds, geographical locations, methodologies, and ruminant species. These genes provide the strongest evidence thus far for significance in association between JD and host genomic variants. Although the proposed candidate genes overlap, the significance of specific polymorphisms vary depending upon population studied, methods, sample size, and phenotypic definitions used. Although imperfect, candidate gene studies provide a strong framework of evidence for genetic influence by these genes in MAP infection.

### SLC11A1

Solute carrier family 11 member 1 gene (*SLC11A1*), formerly natural resistance associated macrophage protein 1 (NRAMP1), is expressed and localized to the lysosomal or endosomal compartment of phagocytic cells including macrophages. This gene product has pleiotropic immunomodulatory effects upon macrophage activation including, increased expression of pro-inflammatory cytokines interleukin 1 beta (IL-1ß) and tumor necrosis factor alpha (TNF-α), increased expression of *MHC-II* gene expression, and prevents bacterial growth within macrophages in early infection (36, 37). *SLC11A1* is one of the most well-studied disease-resistant genes to date, and is associated with resistance to intracellular bacterial infections in mice and humans including; *M. tuberculosis, Mycobacterium bovis, Mycobacterium leprae, Salmonella typhimurium, Leishmania donovani*, and *Brucella abortus* (32, 34, 38, 39). In addition to the immunomodulatory effects elicited by SLC11A1, proteins in this class of ion transporters are vital to maintain the delicate homeostasis within the host cell (37). Mutations resulting in lower expression of *SLC11A1* are associated with increased susceptibility to intracellular infections, in contrast to hosts capable of strong initial expression and subsequent increase of pro-inflammatory signaling to T-Cells to combat invading pathogens (37).

In the case of MAP infection, the role of this gene on the innate susceptibility to infection in mice was documented by Roupie et al. (40). Investigations into the genetic influence and variants thereof in ruminants followed. In cattle, *SLC11A1* gene is mapped to chromosome 2 where the most well-studied polymorphism related to infectious disease resistance/susceptibility is a (GT)n microsatellite polymorphism, characterized by variations in the number of GT repeats (41). This microsatellite polymorphism is located at the 3' untranslated region (UTR) where numerous studies have found significance between these 3' UTR polymorphisms and resistance to *B. abortus* and *M. bovis* infection in cattle and water buffalo (34, 42). These findings were confirmed in JD by Pinedo et al. (36) who found significance between two alleles at this 3' UTR microsatellite locus with susceptibility to JD in a US population consisting of Holstein, Jersey, and Brahman-Angus cross cattle breeds. This study considered any animal positive on any diagnostic test as infected, whereas non-infected animals were negative on all five tests utilized. Another candidate gene study found contradictory results compared to the above, interrogated the entire length of the bovine *SLC11A1* gene for polymorphisms associated with MAP infection in two independent cohorts of European Holstein-Friesian cattle. This case-control study identified positive and negative matches within each subpopulation, using fecal culture as the diagnostic definition of infected/non-infected. Animals in this study were sampled 4 times, where one positive culture indicated a case and negative cultures on all 4 tests were required for negative controls (43). This study found no association for either (GT)n microsatellite polymorphisms in the 3' UTR of *SLC11A1*. Instead, two different polymorphisms both SNPs c.1067C > G, and c.1157-91A > T located at exon 11 and intron 11–12 of *SLC11A1* respectively, were associated with susceptibility to MAP infection in this study population. The intronic SNP c.1157-91A > T, where the A allele was associated with susceptibility to MAP infection is synonymous. Although not resulting in codon changes or direct functional differences in the encoded protein, SNPs within intronic regions can potentially alter regulation and expression of nearby coding genes. The SNP in exon 11 results in a non-synonymous mutation where the C allele associated with susceptibility, resulting in a proline (CCA codon) whereas the G allele results in an alanine at this position (GCA codon) (43). This region of the *SLC11A1* codes for transmembrane domain 8, and a codon change at this position is thought to result in changes in protein structure and binding (43). Despite significance found in the study above, the same non-synonymous SNP in exon 11 was not found to be significant in a recent study of Turkish crossbred and Holstein cattle raised in Turkey (44). Of note, the diagnostic criteria used in the Turkish study was serum ELISA only, which due to its low sensitivity, may have resulted in misclassification of JD status and thus the loss of significance within this population.

In sheep, candidate gene studies in relation to JD are few in comparison to cattle and even goats, although publications on variations of immune responses, pathologies, and clinical severity of disease between breeds indicate strong evidence for a genetic influence in ovine JD (45–47). The study by Reddacliff et al. (48) utilized two flocks of Merino sheep both with JD mortalities at over 10%, where clinical signs, histopathology, fecal culture, tissue culture, serology, and intradermal testing for delayed type hypersensitivity (DTH) were used to determine infection status. In both flocks studied, the microsatellite polymorphism (AC)n located in intron 1 of *SLC11A1* had alleles significantly associated with incidence of disease where the 162 bp *SLC11A1* allele was associated with susceptibility to JD, while the 160 bp allele with resistance (48). Similarly, a candidate gene study in goats from farms with a history of MAP infection found significance between alleles in microsatellite polymorphisms in the 3' UTR of *SLC11A1* with MAP infection. This study found that goats with the B7 allele, (GT)7 had an increased incidence of negative serum ELISA tests (49). A more recent evaluation of SNPs within *SLC11A1* in three goat breeds from India, published agreeable findings in relation to alleles within the A and B polymorphic regions found in the 3' UTR, where of the two alleles in region B, the B7 allele was significantly associated with negative animals compared to the B8 allele at this locus (50). This genotype was also found to be protective in goats to *Brucella* infection, where the B7/B7 genotype was significantly associated with the absence of *Brucella* specific antibodies (33). Further correlating these findings are results from Taka et al. (51) where monocyte-derived macrophages from goats with the B7 genotype at this locus exhibited increased expression of *SLC11A1* and pro-inflammatory cytokine Interleukin 1 *a* (*IL-1a*), which was concluded to be associated with resistance to MAP infection in this study.

Interestingly, polymorphisms identified in human *SLC11A1* have been shown to confer increased susceptibility to infectious diseases such as tuberculosis and inflammatory diseases including Crohn's disease and rheumatoid arthritis (37). Two of these polymorphisms are microsatellite repeats of (GT)n, where allele two at this locus is associated with susceptibility to tuberculosis and reduced expression of SLC11A1 mRNA and increased anti-inflammatory Interleukin 10 (IL-10). In contrast, allele 3 was associated with relative increased expression of *SLC11A1*, and was found to be protective, similar to reports in ruminant species discussed above (37).

### NOD2

Nucleotide-binding oligomerization domain containing gene 2 (*NOD2*) previously *CARD15*, is another highly studied gene in relation to resistance and susceptibility to both inflammatory and infectious diseases. This protein belongs to the family of (Nod)-like receptor (NLRs), a type of pattern recognition receptor (PRR) that recognize and bind pathogen-associated molecular patterns (PAMPs) (52). This leads to downstream activation of innate immune signaling, specifically resulting in canonical Nuclear factor-kappa B (NF-κB) inducing inflammation and subsequent recruitment of lymphocytes to fight infection (52). NLRs are intracellular receptors, thus allowing for detection of PAMPs from intracellular pathogens and are highly expressed in macrophages and dendritic cells (53). NOD2 is as such, and functions to recognize muramyl dipeptide (MDP) derived from peptidoglycan from gram-positive and gram-negative bacteria (52). NOD2 has been found to have increased sensitivity to the *N*-glycolyl form of MDP produced specifically from mycobacterial peptidoglycan, indicating that mycobacterial specific binding to the leucine rich repeat (LRR) domain of this protein influences downstream innate immune activation (54). Previous studies have shown that mutations in *NOD2* inhibit the ability for hosts to recognize and respond to mycobacterial pathogens resulting in increased susceptibility to disease, and has led to investigating the influence of *NOD2* polymorphisms in MAP infection (12, 32).

Candidate gene studies on *NOD2* in cattle have identified conserved regions between the bovine, human, and murine homologs within coding regions of this gene, indicative of a conserved NOD2 function across these species (55). Pinedo et al. (56) identified significance between SNP (C733R) located within the LRR region of *NOD2* and susceptibility to paratuberculosis in a mixed breed cohort of cattle. In contrast, a separate investigation solely focused on the Holstein-Friesian breed found that the SNP identified by Pinedo et al. (56) was monomorphic, having only one major allele in the population and thus no significance was found in this cohort (57). Instead, in this Holstein-Friesian population the C allele of the SNP 1908C > T located at the end of *NOD2* 3' UTR was found to be associated with susceptibility to infection.

To date, there are no candidate gene studies investigating association of *NOD2* polymorphisms in ovine or caprine JD. However, one study found differential expression levels of *NOD2* between asymptomatic, non-infected controls, and sheep with both multibacillary and paucibacillary pathological forms of disease (58). This study found increased expression of *NOD2* in intestinal tissues of sheep with both pathological forms of disease compared to asymptomatic and non-infected control sheep. In this same cohort, a sequence analysis of *NOD2* exon 11 failed to reveal significant associations between disease forms or between non-infected controls and diseased animals. Despite this, one can speculate that if the entirety of the ovine *NOD2* gene had been sequenced significance may have been found (58).

Polymorphisms in NOD2 are some of the most well-documented in relation to susceptibility to CD in humans, and include SNPs within the LRR domain coding region of human *NOD2* (59). The evidence in cattle and humans, as well as the function of this gene product in innate immunity indicate the potential for polymorphisms in ovine *NOD2* to be associated with resistance/susceptibility to Johne's disease.

### TLRs

Toll-like receptors (TLRs) are another class of PRRs similar to NLRs like NOD2, however TLRs are transmembrane receptors compared to the intracellular NLRs, allowing for recognition and binding of both intracellular and extracellular ligands. Mammalian species vary in the number of TLRs, with most sharing up to 11 TLR genes and are imperative for pathogen recognition and initiation of appropriate activation of both innate and subsequently adaptive immunity (53). The most frequently associated TLR genes with susceptibility to MAP are *TLR1, TLR2, TLR4*, and *TLR9*, each responsible for binding variations of PAMPs, where polymorphisms in these genes interfere with the host's capacity to mount immune responses against invading organisms. TLRs 1, 2, and 4 have been implicated in the recognition of mycobacterial components including lipopolysaccharide (TLR4), lipoproteins including lipoarabinomannan, and mycolic acids (TLR2) (60).

Studies in cattle have found significance between multiple SNPs within *TLR1, 2*, and *4* although results for individual SNP significance has been inconsistent. Again, this inconsistency in results between studies is not uncommon in JD literature and will be discussed in detail later in this review. Possible causes for these inconsistencies can include differences in diagnostic methods, breed, number of animals, and statistical models used. A study conducted by Mucha et al. (61) found significance between a SNP within *TLR1* LRR10 region of the ectodomain resulting in a Val220Met amino acid change and an increased incidence of MAP infection in cattle determined via nested PCR. This region of *TLR1* is involved in recognition of microbial lipoproteins, where an amino acid change to methionine at this position can interfere with the formation of the LRR loop structure involved in PAMP recognition (61). Further candidate gene studies in cattle have failed to report significance between the aforementioned SNP or other SNPs within bovine *TLR1* however, the same SNP in the ovine *TLR1* gene was found to be significantly associated with susceptibility to MAP infection down to the exact amino acid position (61, 62). Together this is indicative of mutations in *TLR1* influencing recognition and binding of bacterial lipoproteins, supporting the role of *TLR1* as a potential candidate gene in the susceptibility/resistance to MAP infection in ruminants.

In cattle, SNPs within both *TLR2* and *TLR4* have shown statistical significance in association with MAP infection. A candidate gene study by Mucha et al. (61) identified one significant SNP in *TLR2* resulting in a Ile680Val mutation within the intracellular IL-1R domain, which was not found in a separate candidate gene study. Instead, the SNP TLR2-1903T > C located in the LRR region was found to have a statistically significant association with MAP infection, classified by fecal culture, clinical signs, and ELISA (63). Similarly to bovine *TLR2*, studies have found various SNPs in bovine *TLR4* with conflicting statistical significances between the relative cohort studied. The same study by Mucha et al. (61) found four synonymous SNPs within various locations of the ectodomain of bovine *TLR4* associated with MAP infection from Holstein cattle of Slovakia, but were not detected in other studies on *TLR4* SNPs and resistance/susceptibility to MAP infection in other cattle populations (36, 61, 63, 64).

Less evidence for association between SNPs in ovine *TLR* genes and MAP infection exists, however the few reports seem to support a common role of TLR polymorphisms in differential outcomes of MAP infection. As mentioned above, a significant SNP located within the *LRR10* region of *TLR1* causing a Val200Met mutation specifically at the 9th amino acid position has been identified in MAP infected ovine and bovine animals (61, 62). Mutations at this locus have been shown to hinder the binding and recognition of MAP PAMPs, potentially contributing to increased susceptibility to infection (62). SNPs within ovine *TLR2* TIR domain causing a Phe670Leu mutation were also found to be associated with MAP infection in a population of sheep from Slovakia (62). Recently, a combination retrospective case-control study found a 6.6-fold reduced risk of MAP seropositivity in Turkish ewes harboring one or two copies of the Q650 *TLR2* haplotypes “13,” “15,” and “17,” which also lie within the *TLR2* TIR domain (65). The TIR domain is responsible for the dimerization of *TLR1,2* and *TLR2,6* which promotes protein-protein interactions and influences downstream cytokine and chemokine signaling (66). Evidence for the role of TLR9 in ovine MAP infection was found in a study of sheep from the United Kingdom (U.K), where expression levels of various PRRs from the intestinal tissues between not infected, asymptomatic, paucibacillary, and multibacillary forms of ovine JD were compared (58). All sheep in this study originated from naturally infected flocks, where control and asymptomatic animals lacked symptoms and were either negative for tissue PCR at necropsy (control), or positive (asymptomatic), and diseased animals were further classified using histopathology. This study found significantly increased levels of *TLR9* expression in asymptomatic animals compared to all other groups. This is an interesting finding considering the asymptomatic status of these individuals, as chronicity and persistence of mycobacterial pathogens within hosts is hypothesized to do with sensing of bacterial DNA, not necessarily the living organism (67). The overlap between asymptomatic status, persistence and the role of *TLR9* in recognition of bacterial DNA implicates a role of polymorphisms in *TLR9* to influence resistance/susceptibility to JD in ovine hosts (58).

Polymorphisms in human *TLR2,4* are shown to be associated with susceptibility to both *M. tuberculosis* and *M. leprae* infection in addition to associations to CD (68–70). Specifically, one study identified SNPs in human *TLR4* significantly associated with CD and MAP infection in pediatric patients (71).

### MHC-II

Major histocompatibility complex type II (*MHC-II*) is a complex of genes involved in presentation of antigens to T-cells to induce Interferon-gamma (IFN-y) expression, leading to macrophage activation and subsequent induction of inflammatory responses. MHC-II genes are expressed primarily by phagocytic cells including macrophages, and have been shown to be downregulated in response to MAP infection, similar to other pathogenic mycobacteria including *M. tuberculosis* (72–74). Modulation of this innate host mechanism by MAP delays presentation and subsequent recognition by the adaptive immune system, allowing mycobacterial pathogens time to persist within a protected and controlled microenvironment (75). Polymorphisms within genes belonging to the MHC-II complex have potential to impact not only innate immunity, but also influence activation of the adaptive branch. MHC-II signaling is a known bridge between innate and adaptive immunity, where MHC-II molecules loaded with antigens interact with CD4+ T cells to induce antigen specific immunity in response to infection (62). Mutations that alter the expression, binding, or ability to activate T cells have systemic impacts for the host, leading to inappropriate immune responses that potentially alter the course of infection.

Previous investigations on the influence of *MHC-II DRB3* exon two gene polymorphisms in cattle with production inhibiting diseases including mastitis, have been used as a means of providing potential markers for selective breeding (39, 76). A candidate gene study focused specifically on polymorphisms within *DRB3* exon two region identified six mutations associated with resistance/susceptibility to JD in cattle (76). All mutations were located within the antigen binding site and led to amino acid substitutions which have the potential to interfere with the ability for antigen to bind, thus hindering downstream signaling. Four mutations where associated with susceptibility and two with resistance in this study. Considering the average age (5–6 years) and the average herd prevalence of infection (17–19%), the two polymorphisms associated with resistance may have stronger importance for investigation in future studies (76). In sheep, Reddacliff et al. (48) found significant associations between microsatellite polymorphism alleles in ovine MHC genes and JD in two flocks of fine-wool Merino sheep. Evaluation of disease status was done with both ante and post-mortem tests and found significant association with the 163 allele with susceptibility, and allele 173 with resistance to JD (48). A study conducted by Singh et al. (24) evaluated mutations in the caprine *MHC-II* DRB region with resistance/susceptibility to JD in an endangered Indian breed Jamunapari goats, known to be increasingly susceptible to JD compared to other Indian goat breeds (24). Two di-allelic SNPs were found in this population at the antigen binding site. Significant synergistic effects of the two SNPs on resistance to infection was shown, where the homozygous allele for susceptibility was very strong compared to homozygous resistant allele (24). Recent gene expression profiles from Merino sheep experimentally infected with MAP found that differential expression profiles where “resilient” ewes with enhanced regulation of *MHC-II* DQ alpha and beta genes and decreased expression of *MHC- II DQ alpha 2* (77). The resilient ewes were experimentally infected with MAP, but remained free of infection throughout and at the end of the 2 year 7 month long study where no infection could be found at necropsy using histopathology and culture (77). Mutations within human *MHC-II* genes have been associated with increased susceptibility to leprosy, and tuberculosis (78–80). The importance of *MHC-II* alleles on the formation of microbial communities in the gut has been identified, where mutations can lead to increased susceptibility to infectious and autoimmune diseases, including mutations in human *DRB1* are significantly associated with Crohn's disease (19, 81).

The reviewed candidate genes highlight evidence and species overlap in the vital role of innate immune fitness in the host response to MAP infection, seen in Table 1. Inhibition or interference in pathogen recognition and subsequent induction of host defenses can have detrimental consequences for the host, allowing the invading organism time to establish infection undetected. This delay in innate immune recognition also slows the development of pathogen specific adaptive responses and can set the stage for the course of infection.

**Table 1:** Summary Table of evidence of association in MAP infection by candidate gene and species.

**Gene**	**Location/region**	**Cattle**	**Sheep**	**Goats**
SLC11A1	3' UTR Exon 11 Intron 11–12 Intron 1	(GT)n microsatellite (36) SNP c.1067C>G (43) SNP c.1157-91A>T (43) –	– – – (AC)n microsatellite (48)	(GT)n microsatellite (49, 50) – – –
NOD2	LRR domain	SNP (C733R) (56)	-Expression level changes (58)	–
	3' UTR	SNP 1908C>T (57)	–	–
TLRs	TLR1 TLR2 TLR4 TLR9	Val220Met LRR10 region (61) Ile680Val IL-1R domain (61) SNP TLR2-1903T>C LRR region (63) TLR4 (61) ectodomain –	Val220Met LRR10 region (62) Phe670Leu (62) Q650 Haplotypes (65) TLR9-expression studies (58)	– – – –
MHC-II	DRB Region	DRB exon 2 (antigen binding site) (76)	Microsatellite (48) Expression- (77) DQ alpha 2,	MHC-II DRB antigen binding site (24)

*Evidence for each candidate gene from the discussed studies for cattle, sheep, and goats are below. Overlap of the reported genes, regions, and specific SNPs are evident and provide a framework for future investigations which should focus on studies in sheep and goats. SLC11A1, solute carrier family member 1 gene; NOD2, nucleotide-binding oligomerization domain containing gene 2; TLRs, toll-like receptors; MHC-II, major histocompatibility complex type II*.

## Genome Wide Association Studies

Compared to candidate gene studies, genome wide association studies (GWAS) can identify genetic variants throughout the entire genome instead of focusing on one gene or region to identify potential associations between SNPs and disease. Thus, novel mutations can easily be identified genome-wide and compared between studies of populations, species, and breeds. However, GWAS studies are not without limitations, as the population and sample size heavily influence individual SNP significance. This makes finding markers of low influence more challenging, and may result in the failure to detect SNPs of lower significance (82). SNPs with lower significance may not be causative alone, but function synergistically with other SNPs to influence resistance/susceptibility to MAP infection. Despite the limitations, GWAS studies allow comparison of thousands of SNPs and genomic regions compared to candidate gene studies.

GWAS studies to date have mostly been done in cattle, with the exception of one study from Italy using dairy sheep of the Sarda breed. This aimed to identify candidate genes for resistance in sheep utilizing serum ELISA as the diagnostic criteria (83). This group identified 30 potential genes associated with negative ELISA values within a naturally infected flock, although no significance was found between any of the candidate genes discussed thus far. Despite this, interestingly this work identified one gene within the same positive regulatory domain I containing gene, with zinc fingers (PRDM) gene family as previously identified in cattle (83, 84). Both studies investigated resistance utilizing antibody responses and could indicate an overlap between bovine and ovine MAP antibody response. GWAS studies in cattle predominate, specifically evaluating the Holstein breed compared to one study in Jersey cattle (63, 85–89). The first publication to investigate chromosomal regions or quantitative trait loci (QTL) associated with bovine paratuberculosis was done by Gonda et al. (86) in a case-parental control analysis. This study utilized a large cohort of sires and offspring, and pooled genotyping of positive and negative animals determined by serum ELISA and/or fecal culture results (86). This population of US Holstein cattle was found to have differences between sire families, and identified 8 chromosomal regions associated with increased susceptibility, especially on bovine chromosome 20. Another study on a different population of US Holsteins used the Illumina Bovine SNP chip, to identify SNPs across the genome as markers for susceptibility or resistance to MAP infection (87). This group utilized a variety of case/control classifications due to the unclear mechanisms of disease and how these mechanisms correlate with diagnostic test results. Here, they use MAP culture from tissues, culture from feces, or culture from both sites, and reported varied significances between genomic regions and the case definitions. Two regions of strong association with MAP infection status were found, one located on chromosome 3 and the other on chromosome 9 (87). Association with MAP in tissues was found on a locus of chromosome 3, while the other on chromosome 9 was associated with MAP in the feces the authors correlated this with the possibility that different regions or genes are important at varying stages of infection (87).

Recently, a GWAS study from Gao et al. (90) using two analytical approaches found QTL on BTA 7, BTA 22, and BTA 23 associated with susceptibility to MAP infection in Chinese Holstein cattle. This study utilized serum ELISA values to determine cases (185) and controls (760) and genotyped via Illumina Bovine 50K and GeneSeek HD chips. The identified QTL on BTA 23 overlapped with two from previously reported studies found on the cattle QTLdb submitted from Kirkpatrick et al. (89) and Minozzi et al. (91). This region of BTA 23 is within 1 Mb of tyrosul-DNA phosphodiesterase 2 gene (*TDP2)* which encodes a protein that associated with TNF receptor associated factors (TRAFs) that inhibit NF-κB activation (90). The most significantly associated SNP found in this cohort of cattle was on BTA 22 1 Mb from myeloid differentiation primary response gene 88 (*MyD88*), which encodes an adaptor protein functioning centrally in innate and adaptive immune responses (90).

A study by Pant et al. (92) identified 22 SNPs associated with 12 QTLs, including a QTL in chromosome 7 containing four SNPs each significantly associated with MAP infection designated by serum ELISA in cattle (92). None of the proposed candidate genes or loci identified in this study had been associated previously, however recent studies have found SNPs close to the QTL on BTA7 which was also found to overlap with CD markers highlighting importance of this region (90, 93). One gene within 1 Mb of the QTL on BTA7 was found to have functional relevance to JD pathogenesis. This gene is autophagy-related 10 homolog *ATG10* that functions to mediate interactions between other autophagy related proteins that together function to induce and carryout autophagic functions. Autophagy is a process where host cells recycle organelles, but is also heavily involved in the innate immune defense against a variety of pathogens (34, 94, 95). The importance of autophagy specifically in mycobacterial infection has been identified, where inducing autophagy is shown to reduce mycobacterial persistence in cell culture (70, 73, 96, 97). This is specifically of importance considering the zoonotic aspect of JD, as mutations in multiple autophagy genes are associated with Crohn's disease (98, 99). Further, autophagy proteins directly interact and function together with NOD2, SLC11A1, and VDR, all of which polymorphisms are known in CD, and have been identified as candidate genes in resistance/susceptibility to JD. This interaction and similar genes, and the potential role of SNPs within these genes will be discussed in detail later in this review.

Most genetic association studies in JD have investigated genetics in terms of resistance or susceptibility as the phenotypic definition, however Zanella et al. (100) focused efforts on host tolerance to infection. Tolerance to an infectious disease occurs when a host is capable of dampening the severity of infection, but not resistant to the infection itself. In this study a population of tissue culture MAP positive Holstein cattle were used, where fecal culture was a measure of fitness to determine tolerance. Cases were defined as MAP tissue positive with high levels of viable MAP being shed in feces (non-tolerant), compared to controls that were tissue culture positive but had little to no fecal shedding (tolerant). Analysis was conducted by defining tolerance as both a quantitative and qualitative trait, which resulted in differences in loci identified as significant. The quantitative definition resulted in strong evidence for a SNP on BTA15, and no association was found using the qualitative case-control definition (100). Only 41 animals were involved in the case-control definition of tolerance in this study, which may explain the lack of significant associations found. Despite the lack of significance identified for tolerance, this study highlights the importance of phenotypic definitions in GWAS studies.

The idea of tolerance vs. resistance to infectious diseases in plants has been explored, however less is understood about the dynamics in animal diseases. This difference in defining the relationship between MAP and the host is interesting, considering how a genetic basis of tolerance vs. resistance influences the causative organism. In terms of infection, tolerance has no direct selective effect on the pathogen compared to resistance. In resistance, host genetics select for resistance which can result in the pathogen evolving to circumvent these host defenses by increasing virulence (100). This may explain the latent and highly adapted nature of mycobacterial pathogens, and why these organisms are capable of persisting within the host in the presence of an immune response to the pathogen. Co-evolutionary symbiosis in *M. tuberculosis* infection of humans has been proposed, which may also be a possibility in terms of MAP infection in ruminants. The high number of infected animals relative to the number that progress to disease seen in JD is also seen in human tuberculosis (22). These similar ratios may be indicative of strong mycobacterial evolution with their specific hosts and related to the persistent nature of this genus.

## GWAS Meta-Analysis and GSEA-SNP Studies

Although GWAS methods are powerful at detecting genomic markers of strong significance, markers with low to moderate significance can be lost using this approach. Gene set enrichment analysis (GSEA) can be used to identify candidate genes and associated functional pathways from GWAS SNP data to overcome GWAS limitations. This allows both identification of pathways associated with MAP infection and identification of loci with moderate effects that would be missed with only a GWAS approach (88). This method is also particularly suited for identification of loci associated with complex diseases where multiple genes are likely involved in resistance/susceptibility to disease, such as Crohn's disease and paratuberculosis. Gene Ontology (GO) and Kyoto Encyclopedia of Genes and Genomes (KEGG) are commonly used to identify associated pathways which group known genes together based upon biological functions and gene sets associated with identified significant SNPs from GWAS studies.

In an effort to identify candidate genes and biological pathways associated with previous GWAS data from cattle, one group utilized GSEA-SNP methods and identified one GO set was found to be enriched in association with MAP tissue infection. This GO set named positive “regulation of cell motion,” includes genes involved with the movement of cellular components wherein four new candidate genes were identified each located on different chromosomes (88). These genes (*ENDN2*), (*TDGF1*), (*TGFB2*), and (*PIK3R1*) are located on chromosome 3, 22, 16, and 20, respectively, where chromosome 3 and 20 were already indicated in previous GWAS as potentially harboring QTL for JD resistance/susceptibility. The different loci identified by the GSEA-SNP analysis correlate with the previous notion that various regions or genes may be involved at different stages of infection (87, 88, 101).

One of the most significant GWAS meta-analysis was conducted by Minozzi et al. (91) who combined the SNP data from two previous studies Minozzi et al. (84) and Gonda et al. (86) to further interrogate these data sets to find candidate genes associated with MAP infection (84, 86, 91). Both studies utilized Holstein cattle, with the Minozzi population originating from Italy and the Gonda population from the U.S. Case definitions in each original study varied, and thus two different case control definitions were used for analysis, where serum ELISA or tissue culture results were used. SNPs determined as significant in the independent studies were not found in the meta-analysis, however a common association between QTL on bovine chromosome 9 was seen in both studies. Combined data analysis also revealed novel SNPs on BTA1 and BTA15 associated with antibodies determined by serum ELISA, and tissue infection determined via tissue culture (91). This study is evidence that combining GWAS data can provide increased power to identify SNPs and loci below the significance threshold in the independent studies. Identification of loci associated in both cohorts strongly indicates overlap between populations and pathogenesis of the combined phenotypes, and may uncover stronger associations compared to independent studies alone.

Two important GSEA-SNP analysis studies were conducted to reevaluate the SNP data from GWAS studies, one by Minozzi et al. (84) in Italian Holsteins discussed above, and the other by Kiser et al. (102) in U.S. Holsteins. The first study used serum ELISA as the criteria while the later used tissue infection defined by tissue qPCR or culture, these varied phenotypic definitions contributed to differences in enriched pathways associated with MAP infection upon comparison. The study by Minozzi et al. (84), found only one pathway enriched through GO titled “embryogenesis and morphogensis” and identified potential candidate genes not yet implicated in JD, one of which is located on BTA9. The potential candidate gene identified in this study phosphyloacetlyglucosaminemutase 3 (*PGM3*), is involved in the interaction between mycobacteria and phagocytic receptors in *M. tuberculosis* infection where mutations can lead to reduced survival of mycobacteria within macrophages (103). This finding supports the original association of QTL on bovine chromosome 9 with antibody responses to MAP infection, further implicating this region as significant to host response to MAP infection. In contrast, the GSEA-SNP analysis by Kiser et al. (102), which utilized and compared results between cattle populations in the Pacific Northwest (PNW) and Northeast (NE) regions of the U.S. found multiple enriched GO's associated with MAP tissue infection defined by qPCR for PNW and culture for NE cohorts. Although the specific GO's varied between the analysis of individual populations compared to the combined PNW and NE analysis, all the pathways identified to be associated with MAP tissue infection are related to NF- κB (102). These pathways include Toll pathway, response to oxygen-containing compound, cellular response to hormone stimulus, and response to hormones, where several leading edge genes in each pathway have been previously identified in association to MAP infection. Interestingly, both *TLR4* and *VDR* were implicated as leading edge genes in two and three of the five total GO pathways most enriched for MAP tissue infection. This finding supports evidence for *TLR4* and *VDR* in their role in the immune response to MAP, as candidate genes, and the role of NF-κB in MAP infection (104). The above results also have implications in autophagy, as NF-kB plays a central role in regulation of apoptosis and autophagy. NF-κB and autophagy are also associated with both tuberculosis and leprosy, providing further evidence for the importance of autophagy in MAP infection (102, 104, 105). The study by Kiser et al. (102) also compared leading gene results to those previously identified in tuberculosis, leprosy, and Crohn's disease. They found overlap with two genes, both associated with Crohn's where one gene belongs to the solute carrier superfamily of transport proteins, the same family in which the candidate gene for JD and CD *SLC11A1* belongs (104).

## Discussion

### Functional Mechanisms of Proposed Candidate Genes in MAP Infection

When a ruminant host ingests food or water containing fecal material harboring MAP, the organism is transported through the digestive system until it reaches the small intestine. Here, MAP either translocates from the lumen via microfold cells (M-cells) or through enterocytes into the small intestinal lamina propria where resident antigen presenting cells (APCs) macrophages and dendritic cells phagocytize MAP (106). Once internalized, mycobacterial specific N-glycolyl MDP is recognized and activates NOD2, leading to synergistic interactions with TLRs and TLR heterodimers (TLR2, TLR1, and TLR6) to promote downstream activation of NF-κB signaling and a pro-inflammatory response to infection. NOD2 bound to MDP recruits ATG16L1 to the membrane at site of bacterial entry, and together physically interact to initiate autophagy, promoting pathogen clearance (72). NOD2 expression can also be activated by 1,25D3 bound to VDR receptor, inducing expression of cathelicedin antimicrobial peptide (CAMP) and also initiating autophagic flux (107). Interestingly, in addition to induction of autophagy, CAMP has been shown to colocalize with *M. tuberculosis* in infected macrophages resulting in inhibition of mycobacterial replication and was upregulated in a MAP model of infection in red deer (97, 107–109). Upon MAP internalization SLC11A1 is activated and recruited to the phagosomal membrane, increasing expression of pro-inflammatory cytokines and MHC-II genes to enhance innate responses as well as increased presentation of MAP antigens to adaptive immune system through MHC-II (37). Presentation of MAP antigens via MHC-II to CD4+ T-cells then results in subsequent activation of T-cells, leading to downstream antigen specific cellular and humoral immune responses. The above description is a best case scenario where pro-inflammatory signaling in response to MAP infection is fast and robust, leading to increased IFN-y signaling, efficient induction of autophagy, antigen presentation, and control of MAP infection (110). However, it is well-known that the mechanisms underlying this response are not straight forward. Instead, evidence indicates a continuous complex host-pathogen interplay between the host response to eliminate MAP, and MAP mediated manipulation of these host defense mechanisms. This complex dynamic is further influenced by pressure from SNPs and loci associated with resistance/susceptibility previously mentioned and the downstream effect on the host's ability to produce an effective innate immune response to infection.

Historically, a cellular immune response was thought to be required for protection against mycobacterial diseases, however more recent studies indicate this may not be the case for JD. In paratuberculosis, infected animals progress to clinical disease despite the presence of antigen specific cellular and humoral immune responses that are classically thought as protective (7). In ovine JD the paucibacillary pathology of disease is associated with high CMI responses and increased expression of pro-inflammatory cytokines, yet animals still succumb to infection (58). Why these responses are unable to protect hosts from disease progression is unknown, however several hypotheses exist. One such thought is that this phenomenon is indicative of cellular immune anergy, where antigen specific T cells become less and less reactive to antigen over time (111). Another explanation, is that the early initial innate immune responses determines infection outcome, where vital communication between the innate and adaptive branches are delayed or ineffective via MAP mediated mechanisms and/or by host SNPs. The delayed communication between immune system branches allows MAP time to establish an infection niche, promoting the characteristic chronic infection. Due to the spatial and temporal aspects of this disease, an interruption in initial detection and response to MAP can give the infection an upper hand and have detrimental consequences for the host. A delay in response to MAP results in the host immune system generating antigen specific responses that are too late in the infection process, and thus are incapable of overcoming MAP burden leading to disease (6, 7). In contrast, this means that effective recognition and downstream signaling in response to MAP infection by the innate immune system has the potential to contain infection early on, by initiating adaptive immune responses early in infection. This early robust host response thus has the potential to prevent progression to clinical disease by decreasing the time from MAP exposure to host response. The importance of initial innate immune fitness on outcome of mycobacterial infections has been identified through studies on tuberculosis, where GWAS, candidate gene, and immunological studies have shown the importance of innate immune genes and their function in controlling mycobacterial replication (54, 94, 107, 112). Recently this importance in MAP infection and subsequent disease progression has been eluted to and supported by studies showing the majority of differentially expressed host genes occurs early in infection in cattle, sheep, and red deer (110, 113, 114). This is likely indicative of the host responding to MAP, as well as MAP mediated mechanisms including reduction in *MHC-II* gene expression, modulation of host pro-inflammatory signaling, inhibition of phagolysosomal maturation, and apoptosis (6, 7, 11). This is further supported by GWAS and candidate gene studies in tuberculosis and JD, where the identified genes and/or loci are associated with innate immunity and specifically with the host response to intracellular bacteria. The products of the proposed candidate genes have been shown to function in response to mycobacterial infection, and are interconnected either indirectly through signaling pathways or directly through physical protein-protein interactions (37, 115).

The functional role of specific SNPs and loci may have on innate immune response to mycobacterial infection has been well-studied in the context of CD. *NOD2* polymorphisms within the LRR region associated with CD have been shown to render NOD2 unresponsive to MDP, thus incapable of recognizing and initiating a response against mycobacterial infections (116). *ATG16L1* polymorphisms associated with CD have been shown to cause increased expression of IL-10 and IL-6 compared to wild-type macrophages, and are unable to initiate autophagy leading to pathogen persistence instead of clearance (116). Although the role of autophagy and vitamin D signaling in JD is not well-studied, the influence of both pathways and polymorphisms on *M. tuberculosis* infection is well-documented. Both pathways are interconnected and are shown to be necessary for pathogen clearance, where polymorphisms in autophagy machinery or *VDR* genes predispose hosts to disease (95, 117, 118). Interestingly, Pant et al. (92) identified significance of *ATG10* with QTL on bovine chromosome 7 which participates in formation of the autophagy machinery (92, 119). This association of autophagy genes in QTLs along with the recent identification of VDR as a leading edge gene in a GSEA-SNP analysis in bovine JD, provides evidence that both pathways may also be involved in JD pathogenesis and their roles in infection should be investigated further (102).

A recent study has reported gene-gene epistatic interactions between susceptibility genes for MAP infection in cattle, providing a functional connection between polymorphisms in these genes and MAP susceptibility (120). This study utilized a case-only approach, as the goal was determining if gene-gene interactions exist in affected animals and not the population as a whole. Cases were described as any animal that was positive on any diagnostic test, including ELISA, tissue PCR and culture, and histopathology. Pair-wise allelic models were used to test for interactions between previously identified candidate genes and SNPs, and significance was found especially between *CD209 -TLR4* interactions (120). Other strong interactions found were between *CD209-TLR2, TLR2-TLR4, SP110-SLC11A1, SP110-TLR2, SP110-NOD2*, all of which have physiological relevance to the host response to MAP infection. CD209 is a PRR expressed on dendritic cells (DCs), and is involved with initiation of innate immune signaling to mycobacteria (121, 122). Synergistic and physical interactions between CD209 and the above candidate genes indicates the importance of DC involvement in the initiation and downstream signaling in JD. The strength of the *CD209-TLR4* interaction is significant considering the recognition of mycobacterial LPS by TLR4, and that *M. tuberculosis* and *M. bovis* have been shown to target CD209 to impair DC maturation and induce IL-10 expression (60). This has been thought of as a survival mechanism employed by mycobacteria to generate a microenvironment hospitable for mycobacterial persistence. Although it is unclear if MAP utilizes this or a similar strategy, it is possible considering MAP mediated modulation of the host immune system also includes induction of IL-10 signaling (6). This study also identified interactions between several previously identified candidate genes reviewed here, and provides evidence of interactions in cattle infected with MAP. This work allows further research into more interactions, detailed biological pathways influenced by said interactions, and the role polymorphisms play in this interaction and eventual disease outcome.

The complexities of chronic infectious diseases warrant varied integrative approaches to fully understand pathogenesis. This is true for JD, as there is evidence for breed differences in natural resistance, variations in pathologies, species and environmental differences that together influence MAP infection outcome. This dynamic between environmental factors, and gene interactions may vary with disease state, as MAP functions to circumvent the innate defense mechanisms employed by the host over time. It is the complexity of this evolved relationship between the ruminant host and MAP that requires an integrative approach including aspects of both host and pathogen to understand this interplay. Although great progress has been made in the past decade, there is much more to this paradigm that needs to be understood in order to effectively combat this disease.

### Challenges and Limitations of Genetic Association Studies of Johne's Disease

As with any study, there are limitations and challenges associated with investigations of host genetic resistance/susceptibility to JD. Challenges in genetic association studies include differences in populations, and analysis methods used. In JD, these challenges are further complicated by the chronic nature of MAP and inconsistencies in diagnostic methods used between studies as well as limitations of available diagnostic tests. However, the complex nature of this disease requires an integrative approach to understand mechanisms of protective immunity and studies of host genetics have enabled a more complete picture of the host-pathogen interaction.

Candidate gene studies have enabled identification and verification of loci associated with MAP infection in a variety of populations and host species. These studies are fixed upon one gene or region of the chromosome and thus unable to identify potentially significant associations outside the target region of that study. Utilizing GWAS circumvents this issue, allowing interrogation of the entire genome for markers associated with susceptibility/resistance to MAP infection and disease. This approach has been widely utilized in complex human diseases including CD, however only SNPs with strong associations with phenotype are identified in this method. Thus, in a GWAS study SNPs exhibiting moderate to small associations are lost. Considering the complex nature of JD it is thought that resistance to JD is polygenic, where SNPs with moderate or small associations interacting together may influence pathogenesis compared to a single SNP or a single candidate gene (28). GWAS also does not account for potential epistatic interactions between SNPs, which may limit identification of further gene-gene interactions in resistance to MAP infection (120). Another method discussed in this review is GSEA-SNP analysis, which overcomes the limitations of GWAS in the ability to identify low to moderate strength SNPs. This approach also utilizes pathway analysis methods to determine potential functional associations, allowing for further comparisons between similar conditions and genes within the same functional pathway (104).

Results from genetic association studies vary depending upon the method used, number of individuals, population structure of the study population, geographical region, and breed used in the particular study. In GWAS studies of both tuberculosis and CD in humans had variable results for specific SNPs and loci dependent upon the population studied, where a protective allele in one population may increase susceptibility to the same disease (82, 123). Some genes including *TLR* genes, are highly polymorphic and thus variations between geographical or ethnic backgrounds is common and influences significance (124). For example, allele 2 of the human *SLC11A1* (GT)_n_ microsatellite polymorphism is associated with increased susceptibility to tuberculosis in the African population but significance was not seen in Asian or European populations studied (37). This was also seen between two candidate gene studies investigating NOD2 SNPs, where a SNP identified as significantly associated with MAP infection in one study was found to be monomorphic in another population of cattle (56, 57).

The limitations of genetic association studies mentioned above, combined with the numerous disease specific complexities of JD, magnify the difficulty of investigating host resistance/susceptibility to paratuberculosis. Complicating factors include inconsistent case definition terminology used for disease states, the lack of diagnostic accuracy across spectrum of disease, variations in sample processing methods for MAP detection, and the long asymptomatic stage of MAP infection. From this review, it is evident that changes in case definition terminology can significantly influence the results of genetic association studies in JD, where almost each study utilized different diagnostic methods and case definition criteria. Changing the criteria used for analysis within the same data set has shown to result in identification of different potential candidate genes depending upon the definition (100). Terms used to characterize infection/disease such as infected, asymptomatic, sub-clinical, clinical, diseased, and not-diseased are used in literature with varied definitions based upon diverse diagnostic tests used in each study (10). Differences in diagnostic sensitivities across the time course of disease also influence the outcome of genetic analysis, as does the age of animals used. Due to the chronic nature of MAP infection and extended incubation period, the age of animals studied and the number of sample timepoints used can influence results. Older hosts are more likely to be resistant or tolerant compared to younger hosts where infection hasn't had time to progress. The number of samples used in analysis is also critical, where results from more than one timepoint at least 6 months apart provide increased confidence in case definitions used for analysis.

Likewise, variations in laboratory methods used to detect MAP and diagnose animals was also seen in the reviewed studies. The method of DNA extraction for identification of mycobacteria has shown to heavily influence the success of detection using qPCR (125, 126). The thick waxy MAP cell wall is difficult to lyse, which is even more challenging for clinical samples in a matrix of tissue or feces (125). Differences in extraction methods, such as using one or both an enzymatic and mechanical lysis steps, can determine if MAP will be detected in a clinical sample. This is especially true in asymptomatic infections, especially in ovine JD where MAP load can be low (127). In clinically infected animals the amount of MAP DNA being shed in feces is more than sufficient to detect, however asymptomatic or low-shedding animals may harbor little target DNA and be missed if using protocols only tested on high shedding samples. The length of the intestinal tract also makes it more challenging to find MAP positive tissues especially if no gross evidence of disease is seen. Using combinations of target tissue sections and generating one homogenate to use for testing can help limit the likelihood that MAP will be undetected if present in low numbers.

Although not without limitations, genetic association studies are indispensable tools to investigate complex diseases, where host genetics along with environment likely influence pathogenesis. The applicability of independent genetic association study results are limited, however meta-analysis of additive raw data from multiple studies increases power and strength of association as seen by meta-analysis studies discussed above. Combinations of raw genetic data from hosts of varied geographic backgrounds and breeds could potentially lead to the identification of universal combinations of polymorphisms associated with resistance/susceptibility to MAP infection and enhance the mechanistic understanding of this disease. These combinations of genetic markers can then be utilized by veterinarians and livestock producers to selectively breed for resistance to MAP infection (and/or disease/tolerance) or select against identified susceptibility markers.

## Conclusions and Insights into the Future of Paratuberculosis Research

This work reviewed present evidence for host genetic resistance/susceptibility to paratuberculosis in ruminant hosts, where polymorphisms in innate immunity genes predominated. This along with gene expression studies support the vital importance of innate immune fitness in MAP infection outcome, where early innate immune responses set the stage for the remainder of infection (7, 128, 129). Future studies are needed to determine if early expression of cytokines can be used to confidently predict infection outcome, and if polymorphisms in candidate genes are associated with these differences. Although the exact mechanisms of early INF-*y* vs. IL-10 expression in relation to infection outcome is uncertain, the importance of both cytokines in the delicate balance in MAP-host interactions is clear (7, 129, 130). Future research focusing on the interconnected role of identified polymorphisms and candidate genes and the influence on these cytokines throughout the course of MAP infection is needed. Relationships between host SNPs and early innate immune signaling has the potential to reveal further mechanisms of MAP pathogenesis, and how resistant hosts may maintain this delicate balance of pro vs. anti-inflammatory networks compared to susceptible hosts. Recent cutting-edge work on complex human diseases including CD have revealed interconnected networks of human genes and utilized functional genomics to highlight mechanisms of disease as well as various subtypes and disease severity (131). Similar integrative work in JD is needed to illuminate disease mechanisms associated with differences in infection outcome, and provide a solid framework for future genetic and functional studies in both human and animal MAP infections.

The genetic evidence provided by the reviewed studies supports the observed variation in MAP infection outcome, where the majority of ruminant hosts are capable of containing infection early on and never progress to clinical disease. SNPs associated with the candidate genes *SLC11A1, NOD2, TLR1,2,4,9*, and *MHC-II* are the most consistently proposed polymorphisms in JD genetic association studies, indicating that the functions of these genes are vital in host response to MAP infection. Functional studies have shown that polymorphisms in these genes influence monocyte responses to MAP, altering pro-inflammatory signaling and delaying activation of adaptive immunity (99, 132). GWAS and GSEA-SNP studies have further implicated candidate genes, many of which are interrelated in function to the core common candidates described above. The majority of GWAS findings presented here did not agree in terms of significance level of individual SNPs and loci between populations studied. Numerous reasons for this discrepancy are described above, however despite this the data obtained from these studies is invaluable. Even with the challenges and inconsistencies in individual loci and SNPs identified per study, the candidate genes overlap with those identified for brucellosis, tuberculosis, and Crohn's disease, eluting to similar pathological mechanisms between these diseases (19, 34, 42, 133). Implicated genes function together to initiate an effective anti-MAP response, as well as maintain host cell homeostasis. Given these overlaps, the possibility of autophagy and the vitamin D pathway's influence on MAP infection in ruminants is high. Studies investigating these overlapping genes and functions in JD are needed to determine the role of these pathways in JD pathogenesis and associated polymorphisms (97, 116, 134, 135).

Genetic association studies in the future should aim to utilize consistent and accurate case definitions using diagnostic tests that accurately represent disease or infection state as proposed by Whittington et al. (10). Many of the GWAS studies mentioned utilized serum ELISA as a diagnostic tool, which is useful for indicating potential exposure to MAP. However, ELISA status is not a definitive indication of infection status, as sensitivity varies greatly with disease progression (10, 136). The use of consistent terminology, a combination of diagnostic testing and increased interlaboratory collaborations may result in enhanced agreement between future studies. The need for interdisciplinary collaboration in this field was discussed in full at the 2017 MAP Conference (3) and subsequent consensus document published from this meeting. This collaboration in terms of the potential link between MAP and CD is critical, as results vary extensively based upon methodology used to isolate MAP from human tissue which is difficult under the best circumstances (2, 3, 137). The inability to consistently isolate MAP from tissues of CD patients is one of the unmet criteria for zoonosis according to Koch's Postulates, however collaboration between groups and verification of testing methods may aid in confirming or denying the role of MAP in CD (2, 3, 138). Future efforts in evaluating the zoonotic potential of MAP should also focus on the identified overlapping candidate genes and loci mentioned here, as the similarities in susceptibility to JD and CD further point to an association of MAP and CD.

Investigations focused on host resistance to MAP infection in sheep and goats should also be a future goal, as studies involving cattle predominate, and aligns with recent USDA advances for sheep genomics research (32, 139). This will aid in determining if universal markers exist for JD resistance across ruminant species, or if species variation in host genetics exist, which would indicate varied pathological mechanisms of infection. Once polymorphisms are identified and the functional aspects verified, this information can be utilized for selective breeding to generate a more resistant population over time and thus reducing the economic burden and animal suffering caused by this disease. Although genetic association studies provide insight into the mechanisms of protective immunity to MAP infection, identification of host genome variations is only one side of the story. To fully understand the different mechanisms of MAP-host interactions that occur between various progression states a bi-genomic approach is required. This would allow comparative gene expression of host and pathogen at the same time points, allowing for novel interactions to be identified in animals that remain asymptomatic compared to those that progress to clinical disease.

## Author Contributions

AK reviewed the literature and drafted the manuscript. NS and KP edited and reviewed final work. All authors contributed to the article and approved the submitted version.

## Conflict of Interest

The authors declare that the research was conducted in the absence of any commercial or financial relationships that could be construed as a potential conflict of interest.

## Publisher's Note

All claims expressed in this article are solely those of the authors and do not necessarily represent those of their affiliated organizations, or those of the publisher, the editors and the reviewers. Any product that may be evaluated in this article, or claim that may be made by its manufacturer, is not guaranteed or endorsed by the publisher.
